# Evolving approaches to profiling the microbiome in skin disease

**DOI:** 10.3389/fimmu.2023.1151527

**Published:** 2023-04-04

**Authors:** Yang Chen, Rob Knight, Richard L. Gallo

**Affiliations:** ^1^ Department of Dermatology, University of California San Diego, La Jolla, CA, United States; ^2^ Department of Pediatrics, University of California San Diego, La Jolla, CA, United States; ^3^ Biomedical Sciences Graduate Program, University of California San Diego, La Jolla, CA, United States; ^4^ Department of Computer Science and Engineering, University of California San Diego, La Jolla, CA, United States; ^5^ Department of Bioengineering, University of California San Diego, La Jolla, CA, United States; ^6^ Center for Microbiome Innovation, University of California San Diego, La Jolla, CA, United States

**Keywords:** microbiome and dysbiosis, genomics, metagenomics, next-generation sequencing, atopic dermatitis (AD), acne (acne vulgaris), psoriasis

## Abstract

Despite its harsh and dry environment, human skin is home to diverse microbes, including bacteria, fungi, viruses, and microscopic mites. These microbes form communities that may exist at the skin surface, deeper skin layers, and within microhabitats such as the hair follicle and sweat glands, allowing complex interactions with the host immune system. Imbalances in the skin microbiome, known as dysbiosis, have been linked to various inflammatory skin disorders, including atopic dermatitis, acne, and psoriasis. The roles of abundant commensal bacteria belonging to *Staphylococcus* and *Cutibacterium* taxa and the fungi *Malassezia*, where particular species or strains can benefit the host or cause disease, are increasingly appreciated in skin disorders. Furthermore, recent research suggests that the interactions between microorganisms and the host’s immune system on the skin can have distant and systemic effects on the body, such as on the gut and brain, known as the “skin-gut” or “skin-brain” axes. Studies on the microbiome in skin disease have typically relied on 16S rRNA gene sequencing methods, which cannot provide accurate information about species or strains of microorganisms on the skin. However, advancing technologies, including metagenomics and other functional ‘omic’ approaches, have great potential to provide more comprehensive and detailed information about the skin microbiome in health and disease. Additionally, inter-species and multi-kingdom interactions can cause cascading shifts towards dysbiosis and are crucial but yet-to-be-explored aspects of many skin disorders. Better understanding these complex dynamics will require meta-omic studies complemented with experiments and clinical trials to confirm function. Evolving how we profile the skin microbiome alongside technological advances is essential to exploring such relationships. This review presents the current and emerging methods and their findings for profiling skin microbes to advance our understanding of the microbiome in skin disease.

## 1 Introduction

Human skin acts as a substrate for an ecosystem of diverse life. Serving as our bodies’ physical barrier and largest organ, human skin is home to millions of bacteria, fungi, viruses, and microscopic mites, which collectively compose the “skin microbiome.” Many studies recognize that the combined genomes from the host and microbes, also known as the “hologenome,” determine the complete organism’s health and function. Although human skin is colonized mainly by beneficial microorganisms that share in maintaining skin metabolic processes and act as a frontline defense at our body’s external interface, they and their host all operate in a delicate balance. Microbial dysbiosis, a general term for the disturbed or abnormal distribution, composition, or relative abundance of microorganisms, can influence various local and systemic disease conditions. For example, in dermal layers of the skin, microbes and their gene products directly interact with the host immune system, thereby modulating general skin health (1, 2). Furthermore, growing evidence suggests that microbes from one barrier tissue can alter the activity of distant organ sites between the skin, gut, and brain (3, 4). Thus, skin microbiome studies have the potential to significantly aid our understanding of inflammatory skin disorders (such as atopic dermatitis and psoriasis) and influence wide-ranging pathologies in the same manner that research on the gut microbiome has advanced scientific knowledge of digestive and digestive-related disorders.

Extrinsic and intrinsic differences in skin anatomy and the activity of local cell types drive the variations of both normal and dysbiotic microbial skin communities. As the external barrier from the body, it is constantly bombarded by harsh environmental forces such as water/sun/pollutant exposure, temperature and pH fluctuations, and behavioral influences resulting from hygiene or beauty practices. These constantly changing variables challenge the skin’s ability to adapt and maintain stable and benign/healthy microbiome populations (particularly at the epidermal surface). Additionally, given that skin is our interface, it is important to distinguish the difference between members of the skin microbiome (resident to the skin and holding important functional roles) versus those found on the skin with a transient membership (likely from the environment). Compared to most human microbiomes, the skin’s environment is harsh, arid, and of lower nutrient availability. Thus, by nature, the skin’s ecological habitat limits colonization to a comparatively less diverse subset of organismal taxa than other human microbiome ecosystems, such as the gut. Yet, despite these factors, human skin is host to distinctly different and commonly found microbes belonging to multiple kingdoms (i.e., bacteria, fungi, viruses, etc.) with complex and dynamic community relationships. These resident microorganisms are well-equipped to adapt to the skin, often settling in their preferred niches.

The complexity of human skin and its innate ability to encourage microbial niche specialization can be considered by two major interlinking features. Firstly, skin exhibits a three-dimensional structure akin to a geographical landscape. The average person’s skin surface area is approximately 25 m^2^, making it the largest epithelial surface of the human body, with great opportunity for host-microbe interactions (5). Furthermore, skin thickness varies depending on the body site (from approximately 0.5 mm on the eyelids to 1.5-2.0 mm on the face and 4.0 mm on the heels of the feet). Unlike the intestine, where microbes are typically separated from direct interaction with the body by an antimicrobial mucus layer (6), host cells (including those of the immune system) are in direct contact with microbes, especially those that inhabit deeper dermal layers. Secondly, beneath the surface, the skin contains a complex topology of hair follicles and eccrine, apocrine, and sebaceous glands. Similar to the villous epithelium of the human gut, the skin has approximately five million hair follicles (pores) and sweat ducts whose concave structure and depth significantly increase the complexity and diversity of the skin. The densities of these micro-organs make for skin microenvironments to be broadly grouped into three categories: sebaceous/oily (forehead, scalp, chest, and back), moist/humid (skin around nose and mouth, underarms, elbow bend, abdominal, lower buttocks, back of the knee, and foot), and dry (forearms, back of the elbow, buttocks, and front of the knee/legs) (7), although far more fine-scale variation exists on human skin (8). While these features are universal to the skin, their ability to influence temporal and interpersonal variation creates even more microscale diversity and, thus, microbial community membership patterns (9).

Since the introduction of sequencing-based genetic analysis techniques for identifying microorganisms, there has been a significant increase in our understanding of microbial life on the skin. As evolving genomic technologies have been refined to produce richer microbial datasets, so have the accompanying analytical methods appropriate for skin microbiome studies. This review will discuss new and improved practices for analyzing complex microbial communities on human skin. First, we will overview the current status of genomic and metagenomic-based tools and highlight the emerging statistical and computational methods for analyzing microbiome data while discussing their application to healthy and diseased skin research. Next, we will complement our discussion of current profiling practices with the challenges in exploring microbial communities on the skin. The following section will focus on our current knowledge of the healthy skin microbiome from present studies, given our available tools and their known limitations. Finally, we provide perspectives on a few inflammatory skin diseases, summarizing what we know about the skin microbiome’s role and suggesting opportunities for future research.

## 2 Toolbox for surveying microbial skin communities

**Figure 1:**
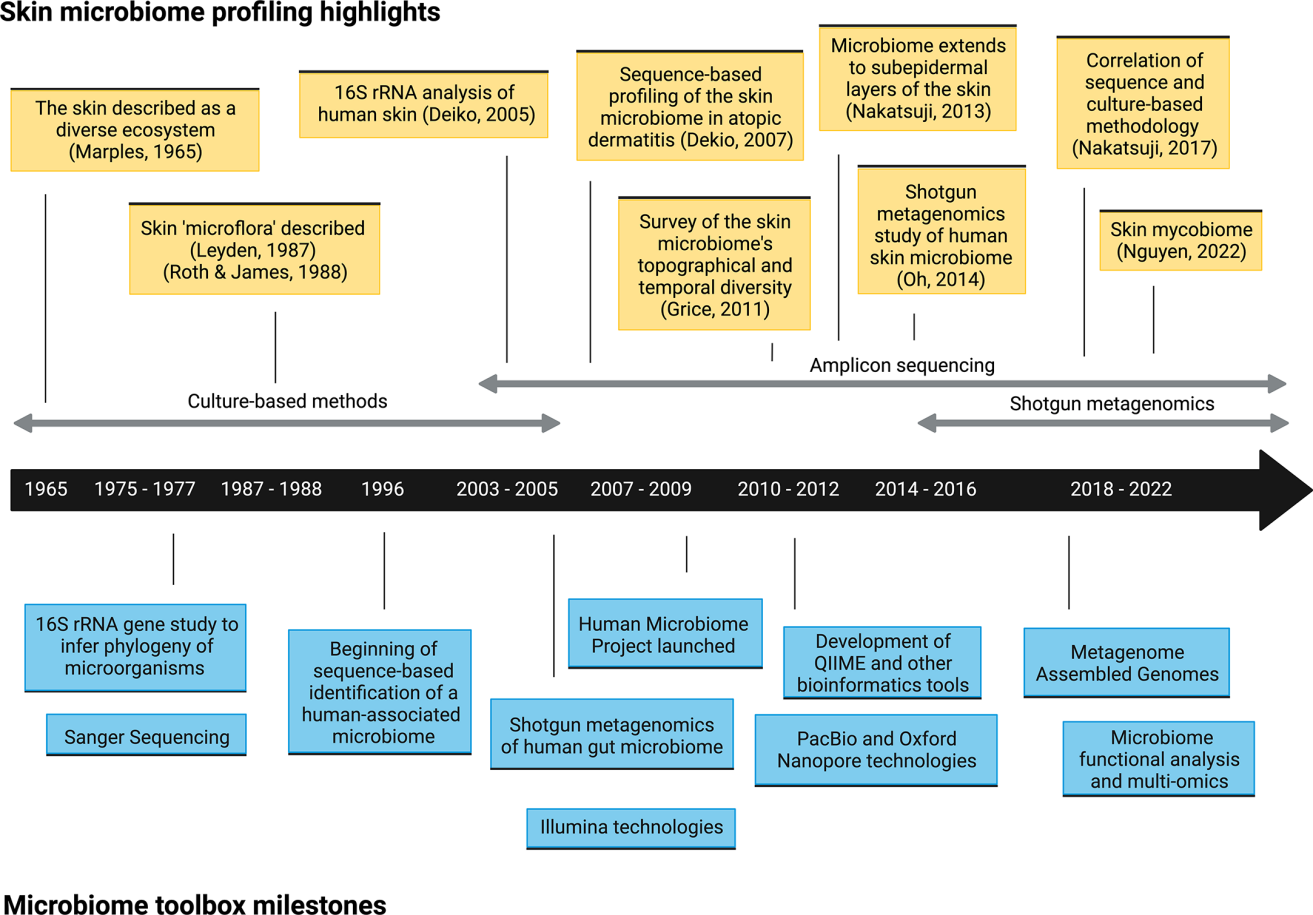
Timeline of selected microbiome toolbox milestones and skin microbiome profiling highlights.

As recently as twenty years ago, culture-based approaches were the only method of exploring microbial communities on human skin (or in any organ) (**Figure 1**). Growing microorganisms sampled from the skin provided critical insights into the skin’s microbial ecosystem. These early pioneering studies identified the major bacterial genera on the skin, including *Staphylococcus*, *Cutibacterium* (formerly *Propionibacterium*), *Corynebacterium*, and fungi such as *Malassezia*, and built the foundation of skin microbiome explorations today (4). However, this practice had a significant limitation; culturing microorganisms *in vitro* inevitably provided biased assessments of microbial diversity. Microbes that grow readily in standard growth conditions overwhelmed those with more fastidious growth conditions, which led to a skewed representation of the community. For example, *Staphylococcus* species are more easily cultured, which led to the underestimation of *Cutibacterium* and *Corynebacterium* populations (10). Furthermore, the culture-based approach limited our window to microbes that we knew how to culture or that persistently survived after removal from the skin, meaning that some could be missed and remain functionally invisible. As technologies for DNA sequencing improved and became more accessible, culture-independent methods, including amplicon-based and metagenomic sequencing, have become the conventional practice for profiling the skin microbiome (**Figure 1**).

### 2.1 Amplicon-based sequencing and analysis

Many studies that survey bacterial skin communities in healthy and diseased states continue to employ targeted amplification, sequencing, and analysis of sampled genetic material to identify microorganisms. This method involves amplifying and sequencing conserved taxonomic markers within prokaryotic kingdoms to resolve genetic variation and inform the evolutionary relationships between organisms. Most of this work has focused on the 16S ribosomal RNA gene (16S rRNA) in bacteria, which continues to be the *de facto* standard for microbial typing, given its ability to identify bacterial taxa. For fungi, the first internal transcribed spacer (ITS1) is the preferred taxonomic marker, whereas 18S rRNA (while still commonly utilized) is far less precise and offers poor discriminatory ability (11, 12). Unlike bacteria and fungi, viruses do not have a common marker gene and are difficult to characterize by amplicon methods.

Although an important and useful approach, amplicon sequencing techniques require careful methodological choices based on prior assumptions regarding the microorganisms in the sample collection. In order to facilitate sequencing of the full-length 16S gene using shorter-read DNA sequencing technologies, which are often limited in their ability to handle sequences of the gene's full length (~1500 base pairs), most protocols utilize specific locations within the 16S RNA gene known as hypervariable regions (V1-V9). These regions are the most variable between taxa and are situated between conserved regions that can function as PCR primers (13). Because hypervariable areas hold the highest nucleotide variability within 16S, targeting them using specific primers and in different combinations minimizes sequencing cost and effort by capturing the most informative taxon-specific variation in a short sequence (14, 15). Certain specific primer pairs have been more widely employed for different environments, and these primers are most often designed to amplify the V1-V3 or V3-V5 regions. While an “optimal” hypervariable region is not necessarily definitive for different environments, PCR primers designed for different broad-level ecosystems are common, as some regions have been deemed more suitable than others based on microbiome type. For example, the Human Microbiome Project has mainly used primers spanning the V1-V3 and V3-V5 hypervariable regions (16), while the Earth Microbiome Project has traditionally used primers targeting the V4 region (17).

Next, the sequence fragments are read, most popularly on the Illumina platform or using long-read technology *via* the PacBio or Oxford Nanopore platforms. A crucial subsequent step involves a bioinformatics-based process to remove spurious sequencing errors and merge reads with the same genetic signals. Historically, the most widely employed method was to cluster sequences into Operational Taxonomic Units (OTUs) that share pairwise sequence similarity (often at a 97% probability threshold) into a representative proxy used for downstream analysis (18). However, this method should not be used today. Improved computational methods, known collectively as oligotyping or grouping into Amplicon Sequence Variants (ASVs) or sub-OTUs (sOTUs), provide substantial improvements relative to OTU picking (19). In contrast to OTU clustering, oligotyping includes positional information from sequencing data by making use of the fact that biologically significant variation is more common at specific positions, whereas sequencing errors are randomly distributed. ASVs use a related approach, assuming that rare variants are often sequencing errors of common members in the population. These approaches resolve more biologically relevant groups at higher discriminatory power. Highly cited bioinformatics tools that are OTU-based include QIIME (20) and Mothur (21), while popular ASV-based tools include QIIME2-Deblur (22, 23) and DADA2 (24). Following OTU or ASV-based analysis, a “feature by sample” OTU or ASV table, providing the count of each feature in each sample, is generated and used for taxonomic assignment. QIIME2 and Mothur both support taxonomic profiling, relying on reference databases. Popular taxonomy databases include Silva (25), Greengenes (26), and RDP (27). Finally, functional profiling using PICRUSt (28) or Tax4Fun (29) can link taxonomic data such as that obtained from marker genes to the predicted biological function of taxa within a community. The significant advances in computational methods of 16S and other marker-gene techniques have greatly improved the accuracy of amplicon-based analyses; thus, some of the first conclusions published regarding the identity of bacterial and fungal species on the skin are no longer deemed accurate or of sufficient resolution, underscoring the importance of making the raw data from studies available for periodic re-analyzation with better techniques.

### 2.2 Shotgun metagenomics and analysis

Shotgun metagenomics, or whole metagenome analysis, attempts to capture and sequence the complete DNA profile of every organism in a sample. This identification method presents an opportunity for a more accurate representation of all microorganisms in a sample and offers multiple advantages over amplicon-based methods regarding the richness of information obtained (30). Multiple systematic comparisons between amplicon sequencing and whole metagenomic sequencing methods have been conducted for the gut microbiome (31–34), although, to the best of our knowledge, none of the skin microbiome. These studies have concluded distinct advantages in using shotgun metagenomics, including identifying a greater diversity of microbial taxa found and enhanced resolution and accuracy of species detection (31, 32), with broad-level biological patterns (such as alpha and beta diversity metrics) being largely consistent (33, 34). Shotgun metagenomics is highly applicable to skin microbiome studies for several reasons. First, this technique can sequence microbes at the whole-genome level, providing a relative abundance of functional genes, not only taxonomic markers. Although marker genes will likely remain an important and efficient way to classify taxa and evaluate microbial communities, shotgun profiling provides a deeper complexity, opening an enhanced window into the microbial dynamics within a skin microbiome sample. Second, because this method is accomplished by randomly fragmenting all DNA within the sample into small pieces, it resolves the sample’s complete DNA profile, including bacteria, viruses, microbial eukaryotes, metagenome elements such as plasmids, and the human host. Thus, whole metagenome analyses in the skin microbiome context can obtain multi-kingdom genomic and metagenomic signatures, extending past the phylogenetically limited views of marker gene analyses. Third, because this method resolves whole genomes, shotgun metagenomics provides higher phylogenetic resolution and is better equipped to differentiate species and strains (provided the sequencing is deep enough and the assembly sufficiently successful) (35). As mentioned previously, human skin is preferentially dominated by many different *Staphylococcus* species (*S. epidermidis*, *S. hominis*, and *S. lugdenensis*, to name only a few), which makes short-read amplicon-based genus-level taxonomic resolution limiting, and whole metagenome approaches more capable of discriminating this important taxon.

Illumina platforms are the most popularly employed technology for short-read shotgun metagenomics, followed by Ion Torrent. Long-read machines from Oxford Nanopore MinION and PacBio technologies are scaling rapidly and are likely to see more significant usage. Following sequencing and necessary quality filtering and trimming steps, metagenomics workflows require linking fragmented reads to taxonomic and functional information. Two major approaches carry out this process: reference/read-based and assembly/contig-based workflows (30). Reference-based approaches to identifying microorganisms involve mapping sequences and comparing them to reference genomes from relevant databases. Assembly-based approaches attempt to reconstruct fragmented reads representing the community of microorganisms into contigs and then group or “bin” contigs into species, sometimes referred to as Metagenome-assembled genomes (MAGs) (36). The *de novo* or reference-free approach of MAGs allows for more comprehensive identification of skin microorganisms, including those previously uncharacterized, thus expanding our knowledge and accuracy of the skin’s microbial inhabitants (37).

### 2.3 Integrating other ‘omics’ technologies for functional studies

For a complete understanding of the composition and function of microbial communities, holistic approaches that go beyond marker gene analysis and metagenomics are necessary to begin inferring the functional roles of different microorganisms. Metatranscriptomics involves sequencing the RNA profile of microorganisms in an environment, thus enabling profiling of which genes are being expressed at a specific time and how they are regulated. Additionally, due to the shorter half-life of RNA, transcriptomic analysis has some ability to distinguish between live or dead microorganisms and can capture a more dynamic community profile. Combining metatranscriptomics with marker gene analysis or shotgun metagenomics can improve understanding of which microorganisms in a community are actively transcribing and their functional role. Likewise, metaproteomics and metabolomic approaches seek to characterize the proteins and small molecules consumed and produced by the microorganisms to validate profiling findings. For example, 16S sequencing combined with mass spectrometry corroborated the hypothesis that *C. acnes* utilized sebum triglycerides as an energy source by showing their colocalization with fatty acid products (38). A recent study by Roux et al. described the existence of three distinct metabolite-microbe clusters at the skin surface in infants that parallels our current view of the three major environments on human skin; one characterized by *C. acnes* and sebaceous elements at the epidermal barrier, another microbiome network involving *Staphylococcus* spp., moisture and pH regulation at the skin surface, and a third niche with a diverse but unique metabolomic profile which was *Streptococcus*-centered (39). Critically, however, these already complex data sets must be integrated with companion functional data and analysis of the human host to understand how microbes contribute to the health and disease of the “holobiont” (40). Analysis of multi-omics data from the skin microbiome will provide a more complete picture of the behaviors of skin microbes. Furthermore, machine learning and artificial intelligence methods are increasingly being applied to skin microbiome research, including determining changes in abundance or diversity of species and strains, integrating multi-omic microbiome data, and phenotypic prediction (41–43).

### 2.4 Statistical considerations for microbiome studies

Microbiome data analysis, which typically involves comparing a matrix of features composed of taxa or genes from a simple case to samples of different experimental or phenotypic groups, can be quite complex. Therefore, it is important to use caution when interpreting the results of statistical analyses. Even seemingly basic questions, such as “how does a sample from diseased skin differ from that of healthy skin?” often require complex statistical interpretation. Microbiome data is multi-dimensional, meaning it has a large number of features that can quickly increase with the number of microbes, samples, and time points. Additionally, such data is typically very sparse, with most microbes not present in most of the data, creating a significant skew towards the zeros of the dataset, which represent virtually non-existent microorganisms. Microbiome datasets are also compositional (44), meaning that the sequence counts are heavily influenced by the limitations of the sequencing technology, not just the actual abundance of microbes in the sample (35). These characteristics make it difficult to interpret microbiome data, requiring appropriate and validated techniques.

Microbiome datasets and their broad-level patterns are commonly evaluated using alpha and beta diversity metrics. Alpha diversity measures feature diversity (i.e., taxa or genes) within a single sample and can be compared between different groups of samples. Alpha diversity analyses may describe the diversity of microbial genera or species between skin disease and healthy groups. In contrast, beta diversity measures the difference in composition between two communities. Traditional statistical tests such as Student’s t-test or ANOVA should not be used to compare diversity or the relative abundance of taxa across samples because the data often does not follow a normal statistical distribution and/or violates independence assumptions (18). Instead, non-parametric tests should be used to avoid high false discovery rates. Typically, statistical evaluations use the Mann-Whitney U test or Wilcoxon rank-sum test instead of the t-test, Kruskal-Wallis or PERMANOVA instead of ANOVA, and Spearman rank correlation over Pearson correlation. Newer, compositionally aware tools are especially important for differential abundance comparisons. Effect size calculations provide the magnitude of differences between groups and offer a more quantitative understanding of statistical significance beyond p-values (45, 46). For a more extensive review of the quantitative tools and techniques for microbiome data analysis and visualization, we direct the reader to these reviews (18, 47).

## 3 Special considerations in the sequencing and analysis of skin microbial communities

### 3.1 Challenges due to low microbial biomass

Whether pursuing culture-based, amplicon, or shotgun genomics methods, there are many challenges involved with analyzing the skin microbiome due to the skin’s unique features that can lead to poor-quality genomic data and difficult downstream bioinformatics analysis. Standards and best practices for conducting skin microbiome studies have been described primarily for 16S rRNA gene amplicon sequencing (48) but are largely lacking for metagenomics. Compared to the rich environment inhabited by gut microbes, the skin microbiome holds relatively low microbial biomass (especially at the surface) due to its dry and nutrient-poor conditions (49). Thus, it is critical to note that microbial DNA from skin samples is particularly prone to dilution from human and environmental DNA, and 90% of sequencing reads can be from human DNA when applying metagenomics (50). Furthermore, the issue of low microbial biomass makes it difficult to identify rare or naturally low-abundant taxa that may still have clinical relevance and distinguish these from transient skin microorganisms or possible environmental contaminants. Additionally, characterizing non-bacterial DNA, particularly viruses, whose genomes and microbial biomass are magnitudes smaller than bacteria, presents even greater challenges.

Deeper metagenomic sequencing mitigates issues in low microbial biomass; however, this, in conjunction with appropriate DNA extraction and host/free microbial DNA depletion methodology for skin microbiome samples, is likely the most ideal and cost-effective. Reduction in human DNA during the DNA extraction and library preparation process for skin samples is possible and seems to be better performed by some commercial kits than others (50). However, to the best of our knowledge, there are no published studies that apply skin-specific methods for a host, bacterial extracellular DNA, or dead bacterial intracellular DNA depletion for the skin microbiome. Chemical digestion or lysing to deplete excessive host DNA after DNA extraction may be necessary before microbial sequencing proceeds from skin samples, as demonstrated for the oral and nasopharynx microbiomes (51, 52). Likewise, a method for depleting both human and free microbial extracellular DNA for metagenomic sequencing *via* selective lysis of eukaryotic cells and endonuclease digestion has been proposed for sputum samples from the Cystic Fibrosis respiratory microbiome (53). Here the authors noted significant increases in microbial sequencing depth and detected taxa and genes compared to commercially available kits. Careful storage procedures of microbial DNA from skin samples are also necessary to prevent further degradation (54). Due to the low abundance of microbial DNA on the skin, the environmental contamination risk is high. Thus, it is crucial to use negative/blank controls throughout all processing steps, from DNA extraction to library preparation and sequencing in parallel with experimental samples. Mock communities (artificial mixtures of known microorganisms) and positive/reference samples are also necessary to benchmark results.

### 3.2 Challenges due to sampling technique

Human skin represents a vast, multi-layered three-dimensional organ that spans the entire bodily surface. Body-site variation and physiological anatomy create distinct environments, allowing for specialized microbial colonization and unique niche microbiomes. Thus, where you sample on the body is considered to heavily influence results. As the skin contains many layers and species are unevenly distributed, the sampling technique will strongly influence the results (55). Most human skin microbiota studies use swabs as the sampling method; however, this practice is limited to collecting surface and epidermal skin microbes (2). In contrast, a pore strip sample captures mostly microbes in the follicular environment, including the hair follicle and sweat duct. Other sampling methodologies, such as skin biopsies, examine the microbial community within follicular and deeper dermal environments; surface swabs do not always reach those areas. An analysis comparing epidermal and dermal sampling methods suggests the dermal compartment has a greater abundance of microbes with less variability between anatomic locations and between individuals, termed the “universal dermal microbiome” (2). While such observations challenge current research assumptions, this finding may not be surprising as environmental influences (including the individual’s hygiene and cosmetic practices) affect the skin microbiome, particularly at the surface. However, so far, fewer studies have performed dermal sampling as it is more invasive. Additionally, performing a skin scrape, biopsy, or other more invasive sampling procedure may capture an even larger proportion of host (human) DNA contamination, posing an additional challenge when choosing this approach.

### 3.3 Challenges of amplicon methods

Skin microbiome surveys are strongly influenced by the analytical method, including the choice of hypervariable region for 16S-based studies (55). According to Meisel et al., sequencing V1-V3 of skin organisms provides similar results and is more optimal than V4, one of the most commonly employed sequencing targets for investigating the human gut microbiome. Sequencing of the V4 poorly captures *Cutibacterium* and cannot reliably speciate *Staphylococcus* (55). *Cutibacterium acnes* and *Staphylococcus* species are among the most prevalent commensal bacteria, so this presents a significant limitation in the usability of the V4 region for microbes inhabiting the skin. Given that the V4 hypervariable region is shorter and contains more sequence conservation (56), its difficulty in speciating *Staphylococcus* was unsurprising. These findings highlight how the optimal choice of the most useful hypervariable region is ecosystem dependent and can affect results. Improved 16S rRNA V4 gene primers have been designed to specifically improve the capture of underrepresented taxa (57); however, specific benchmarking for skin bacterial communities and identification ability towards *Cutibacterium* and *Staphylococcus* species has not been validated. Importantly, no single hypervariable region nor the whole 16S gene can distinguish all bacterial species for any human microbiome. Only genus-level resolution is typically reliable when using short 16S regions as a marker, although publications often report species-level resolution without checking whether it is even possible for the relevant 16S region and taxa (resolution varies in different parts of the phylogenetic tree). Consolidating OTUs after sequencing is problematic for two major reasons also. Firstly, selecting one OTU sequence as a proxy tends to ignore minor genetic variants and oversimplify the dataset (58). Second, the canonical 97% clustering threshold is outdated and is now believed to be too low for reliable differentiation for most species (59). Originally proposed in 1994, this metric has been based on multiple linking proxies: short-read sequencing of hypervariable regions to approximate full-length sequence identity and full-length sequence identity to approximate whole genome similarity. Thus, reference libraries created by 16S rRNA gene sequencing approaches require systematic re-evaluation now that whole genome sequencing has produced far richer datasets. Barnes et al. investigated the OTU *vs*. ASV method in the atopic dermatitis skin microbiome, particularly those assigned to *Staphylococcus* species (24). Their findings suggested that OTU clustering inflated bacterial richness and concluded the ASV approach with DADA2 managed sequencing errors better. However, the authors stress that when using amplicon sequencing, one should be careful not to overinterpret taxonomic calls at the species level for the *Staphylococcus* community. Genus-level resolution is possible, but attempting to distinguish between species by sequencing only the 16S gene should be treated carefully.

The next step involves taxonomic assignment based on the homology of sequence reads to reference databases. As 16S rRNA sequencing has been widely used, many well-curated databases are available for taxonomic identification. However, careful considerations regarding taxonomic classification are still required. While it has been suggested that ecosystem-specific databases provide more accurate taxonomic classifications (60), this is likely not true for the skin microbiome. General-purpose microbial taxonomic databases outperform habitat-specific databases in microbiome datasets with significant environmental taxa since these reads may be erroneously assigned if an appropriate reference genome is unavailable (26). Instead, applying environment-specific taxonomic weights within general-purpose databases, such as the Greengenes 16S database with q2-clawback (61), can improve sequence taxonomy classification accuracy from common environments.

### 3.4 Challenges of whole metagenome methods

Shotgun metagenomics has the potential to yield rich and informative datasets. However, as our technology has advanced, so have the accompanying complexities with data analysis. This technology is still relatively more expensive (though widely becoming more accessible and less costly) than amplicon methods. Producing datasets with sufficiently rich sequencing depths (the number of reads per sample) across the whole genome is crucial relative to other methods, especially when the sample is mostly composed of host DNA reads (50). While shallow- or moderate-depth sequencing has been shown to accurately obtain species-level information, particularly in large-scale studies where deep sequencing is cost-prohibitive, only at deep sequencing depths is the attractive lure of strain-level taxonomic assignment and identification of novel strains and low-abundance taxa with biologically meaningful function within the community possible (35). In skin microbiome studies, the potential to distinguish between *Staphylococcus* species and other commensal strains on the skin is only legitimate if sufficient sequencing depth is achieved. This remains a critical problem because skin sampling typically provides lower biomass of microbes than other body habitats, and low-depth data is common. Contamination from host-derived DNA is a constant challenge, particularly in areas where host DNA dominates microbial DNA, such as the skin. When conducting shotgun metagenomics studies on the human skin microbiome, it is important to filter out sequences from the host. However, the amount of DNA needed for shotgun studies has steadily decreased as the technology has improved, allowing for more efficient sequencing of skin microbiomes.

In contrast, as a newer technology, shotgun metagenome databases currently lack many full reference genomes of microbes and are currently most well-positioned for gut microbiome studies. With the advent of whole metagenomics technology, previous skin microbiome studies observed significant metagenomic reads that did not match those in reference databases suggesting unmapped prokaryotic diversity (62, 63). Thus, the *de novo* (reference-free) approach of MAGs and improved assembly algorithms provide avenues to characterize new and unknown skin species from all domains of life. To address this gap, cultivating microbes to provide high-quality whole-genome sequences, which can then be aligned with MAGs, can provide a more comprehensive catalog of microbial diversity on the skin. This integrated approach has been launched by the Skin Microbial Genome Collection and is expanding our understanding of skin microbes to include new bacterial and eukaryotic species and improved assemblies of viruses and jumbo phages (37).

## 4 Present knowledge of the healthy skin microbiome

**Figure 2:**
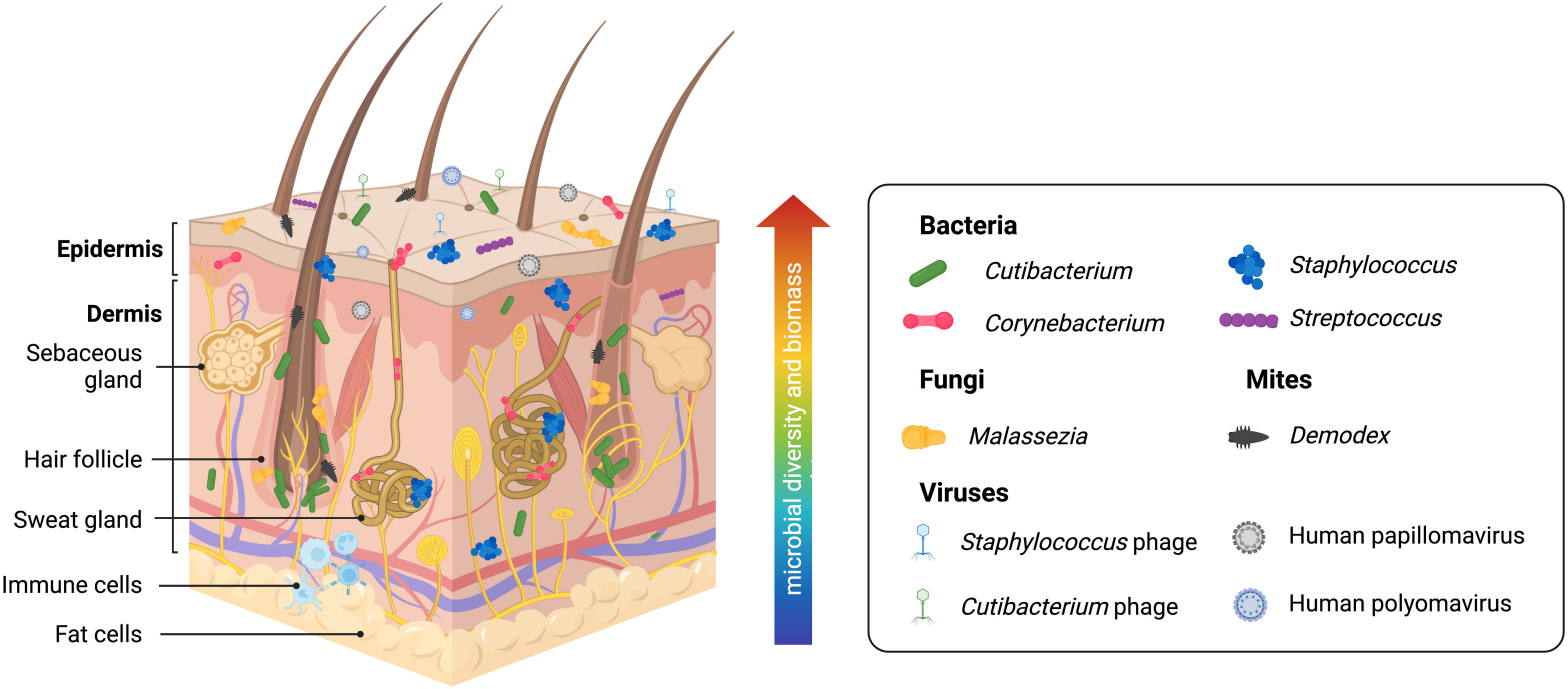
Healthy human skin is host to a diverse range of microbes across multiple skin layers and microenvironments. Skin bacteria, many of which belong to the *Cutibacterium*, *Staphylococcus*, *Corynebacterium*, and *Streptococcus* genera, are resident members of the skin microbiome. The skin eukaryome commonly includes fungi and microscopic mites belonging to the *Malassezia* and *Demodex* genera, respectively. Viruses, most notably including *Staphylococcus* phages, *Cutibacterium* phages, human Papillomaviruses, and human Polyomaviruses, are among the dominant members of the skin virome. Archaea (not pictured) has also been identified on the skin microbiome, though likely with a minor or transient presence. Microbial diversity and biomass are typically higher at the skin surface. However, human skin is a three-dimensional space, and microbes form communities that live at the skin surface, deeper skin layers, and within microhabitats such as the hair follicle and sweat glands, allowing complex inter-species relationships and interactions with the host immune system.

Understanding normal microbiota and typical/benign variations within the skin microbiome in health is necessary to establish a point of reference for investigations into dysbiotic disease states. Human skin contains many resident commensal microorganisms, including bacteria, fungi, viruses, and mites, that utilize skin resources while playing a central role in skin homeostasis and health (**Figure 2**). However, even commensal bacteria can become pathogenic in specific contexts and contribute to inflammatory skin diseases. The following will summarize current information on this topic. However, it is important to recognize that many reports are subject to the limitations of the sequencing technology employed or the sampling methods used. Thus, some revision of current conclusions will likely occur as better data is developed.

Skin microenvironments are broadly grouped into three categories due to the densities of hair follicles and sweat glands: sebaceous/oily, moist/humid, and dry (7) (although far more fine-scale variation exists on human skin) that is preferentially dominated by commensal organisms. Current data suggests sebaceous body sites, particularly within the follicular microenvironments, are the skin sites showing the least species diversity due to being dominated by *Cutibacterium* species, particularly *C. acnes* (64, 65). Interestingly, a recent study suggests that skin pores spatially segregate *C. acnes* genotypes and that each pore on the face is dominated by a population of *C. acnes* typically differing by < 1 mutation (66). Thus, microenvironments within the skin, such as the hair follicle, represent opportunities for population bottleneck effects. Within most moist areas on the epidermal surface, *Staphylococcus* and *Corynebacterium* species are the most abundant (10, 65). Dry sites have the greatest diversity with a mixed population of *Cutibacterium*, *Staphylococcus*, *Corynebacterium*, and *Streptococcus* species and host more transient microbes (65). *Staphylococcus* commensal species are highly abundant across all skin sites due to the diversity of this genus and their facultative anaerobic abilities; these most commonly include *S. epidermidis*, *S. capitis*, *S. hominis*, *S. lugdengensis*, *S. haemolyticus*, and *S. warneri* (65). Human skin also contains a diverse fungal microbiome (mycobiome) that has only begun to be explored by sequence-based methods (67). *Malassezia* species, particularly *M. restricta*, *M. globosa*, and *M. sympodialis*, are the most prevalent fungal microorganisms on human skin, particularly within the sebaceous sites (65, 67). *Candida* species, including *C. albicans* and *C. auris*, are opportunistic fungal pathogens that frequently colonize the skin (67). The human viral microbiome (virome) is also vastly under-characterized for similar reasons as the human skin mycobiome. Bacteriophages (viruses that infect bacteria) are significant members of the microbial ecosystem on the skin. *Cutibacterium* and *Staphylococcus* phages are found to be conserved across individuals. However, little is known about the interactions and dynamics between commensal bacteria and bacteriophages on human skin. Eukaryotic viruses, some of which include human papillomaviruses and polyomaviruses (including Merkel cell polyomaviruses associated with skin cancer), have also been found on human skin (62, 65, 68, 69), and they are believed to be more specific to individuals than conserved in certain anatomical sites. Technical limitations with amplicon sequencing and shotgun metagenomics complicate studying the skin virome. Viral genomes lack a taxonomic marker gene like 16S for bacteria or ITS1 for fungi needed for amplicon sequencing and are easily overwhelmed by prokaryotic or eukaryotic genomes (present in populations that are orders of magnitude larger) when shotgun metagenomics is applied (70). Also found on the skin are *Demodex* mites, microscopic arthropods that are a common (and usually benign) part of the commensal skin microbiome (71–73). These mites prefer sebaceous skin sites as their main food source is sebum. Thus, they have been primarily found in the face and scalp, with greater abundance in the pilosebaceous unit. Two *Demodex* species have been identified so far on human skin, *D. follicularum* and *D. brevis*, with imbalances linked to rosacea (71). Though most overlooked, archaea have also been found to be a part of the skin microbiome (74–77). While there is a general lack of consensus on the abundance and role of archaea on the skin, these 16S gene signatures are reported to belong to the *Thermoproteota* (formerly *Thaumarchaeota*), *Methanobacteriota*, and *Halobacteriota* phyla (74–77). Ammonia-oxidizing *Thaumarchaeota* has been suggested to contribute to lowering skin pH and, thus, supporting the skin’s barrier against foreign and pathogenic microorganisms (74). In contrast, a recent study profiling skin archaeal communities shows that archaea comprise less than 1% of mammalian skin samples and are more likely to have a minor and transient presence driven by the environment (76). Commonly touched surfaces that interface with the skin, including computer keyboards, phones, and door handles, contained archaea, highlighting how our built environments contribute to microbial inhabitance on the skin (76).

## 5 The skin microbiome in inflammatory skin diseases

Research in the microbiota of other organs has shown that subtle differences in genetic and metabolic diversity can drive otherwise commensal microbes toward pathogenesis, defining these organisms as pathobionts. These alterations in community composition can have significant implications for disease onset and treatment. While progress has been made in identifying the different types of microbes associated with various skin diseases, whether a dysregulated microbiome causes inflammation or if these associations are a byproduct of the inflammatory environment is unclear (to varying degrees) for different skin diseases. Inflammatory skin disorders have multifactorial etiologies where an intimate balance of host determinants, environmental influences, and microbial composition can influence disease development. Most microbiome studies of inflammatory skin disorders have utilized 16S amplicon sequencing to investigate microbial alterations with specific skin diseases, illuminating important findings. However, deciphering only bacterial genera and their relative abundance has limited usefulness for expanding our current understanding of skin diseases toward improved clinical application. Understanding differences in strain behaviors that could cause them to be pathobionts, commensals, or truly beneficial mutuals may become possible as more comprehensive genomic methods are brought to bear. This is becoming apparent as more studies are applying shotgun metagenomics to the microbiome in skin various diseases (**Table 1**). Likewise, it is becoming clear that inter-species and inter-kingdom interactions between skin microorganisms play a significant role in skin health as well. Understanding how skin-disease associated microbes interact with the immune system allows opportunities for a deeper understanding of skin microbiota. Such insights will yield powerful and actionable insights into skin disease mechanisms that experiments can validate to confirm function and translate to clinical application. Here, we will focus on areas showing the most progress or best evidence regarding the microbiome’s role in inflammatory skin diseases given currently employed methodologies.

**Table 1:** Recent studies investigating the microbiome in skin disease utilizing whole metagenomics.

Disease	Whole metagenome studies	Bacterial alterations	Fungal alterations	Other alterations	Key remarks
**Atopic Dermatitis**	Bjerre et al. (78)	*S. aureus* abundance was higher in all skin sites except from the feet. In the flexures, *S. epidermidis* colonization accompanied *S. aureus* dominance. Decreased *S. hominis* and *C. acnes* was observed where *S. aureus* was highly abundant	Increased *M. osloensis* and *M. luteus* in all AD skin sites except for the neck, where it was absent	Increased abundance of *Cutibacterium* phages PHL041 and PHL092 and *S. epidermidis* phages CNPH82 and PH15 in AD	Dysbiosis in AD is global and site-specific, involving bacteria, fungi, and viruses
	Byrd et al. (79)	↑ *S. aureus* in severe AD ↑ *S. epidermidis* in less severe AD At strain-level, *S. aureus* populations were clonal while *S. epidermidis* were heterogeneous	The authors stated fungal and viral communities were not significantly different in this study likely due to limited reference databases/genomes		Strain-level functional differences contribute to the complexity of AD
**Seborrheic Dermatitis**	Saxena et al. (80)	↑ *S. epidermidis* ↓ *P. nitroreducens* ↓ *C. acnes*/*S. epidermidis* ratio	↑ *M. restricta*/*M. globosa* ratio ↑ uncultured *Malassezia spp*. ↓ *M. globosa*	Not applicable	The SD scalp microbiome is associated with common bacterial and fungal commensals, and uncharacterized *Malassezia* species
**Acne vulgaris**	Barnard et al. (81)	↑ *C. acnes*/*C. granulosum* ratio	Not applicable	↓ *C. acnes* phage	A balance of metagenomic elements shapes the skin microbiome in acne and health
**Psoriasis**	Tett et al. (82)	*↑ Staphylococcus* with no significant differences at species-level	Potentially, *M. restricta*, *M. globosa*, and unknown *Malassezia spp.*	Not applicable	Strain-level variations could be key determinants of the psoriatic microbiome. Uncharacterized *Malassezia* species were also found in the skin mycobiome
**Rosacea**	None				
**Hidradenitis Supprativa**	None				
**Netherton's Syndrome**	Williams et al. (83)	↑ .*S. aureus* ↑ *S. epidermidis*	Not applicable	Not applicable	A monogenic disorder in a protease alters microbial community composition and promotes inflammation

The up arrow means increased, and the down arrow means decreased.

### 5.1 Atopic dermatitis

Atopic dermatitis (AD), also known as eczema, is a chronic inflammatory skin disease characterized by itchy, red, swollen, and cracked skin flares affecting up to 20% of children and 3% of adults (84). Compared to other dermatological disorders, the role of skin microorganisms and dysbiosis in adults with AD is well-documented and exhibits the clearest relationship in which a dysregulated microbiome is causative to skin inflammation. The skin microbiome of adult AD patients typically has reduced biodiversity due to a massive increase in *Staphylococcus aureus* colonization which precedes lesion onset (85, 86). Likewise, skin colonization by *S. aureus* has been found to precede AD diagnosis in infants (87). The dominance and overgrowth of *S. aureus* result in a relative decrease in the proportion of commensal bacteria belonging to the *Cutibacterium*, *Corynebacterium*, and *Streptococcus* genera (85, 88). *S. aureus* commonly presents as a skin pathogen, and colonization by *S. aureus* on healthy skin is rare (89). One recent shotgun metagenomics study by Byrd et al. suggests that not all *S. aureus* strains may be equally pathological (79). This initial work provides insights into *S. aureus* strains from lesional versus non-lesional skin on AD patients. *S. aureus* strains from lesional versus non-lesional sites are phylogenetically clustered separately, suggesting that strain differences may be implicated in AD severity. Multiple studies provide functional evidence of *S. aureus* producing toxins, proteases, phenol-soluble modulins (PSMs), bacteriocins, and other virulence factors exacerbating skin inflammation (90–95).

However, despite a clear link between *S. aureus* and AD, not all AD lesions are colonized by *S. aureus*; many AD patients also have greater colonization of *S. epidermidis* than *S. aureus* (85, 96, 97). These observations are also noted in studies of the AD microbiome where *S. aureus* exclusion leads to greater *S. epidermidis* dominance. Findings from a recent metagenomic study suggest *S. epidermidis* may be a secondary causative agent of AD disease severity as *S. aureus* significantly associates with severe disease, while *S. epidermidis* may be more common with moderate disease (79). Compared to *S. aureus*, *S. epidermidis* is universal on human skin, and some strains exhibit many beneficial properties for skin health, like defense against opportunistic pathogens and enhancing epithelial barrier function (98). For example, *S. epidermidis* induces antimicrobial peptides from keratinocytes and produces phenol-soluble modulins (PSMγ and PSMδ) and other small antimicrobials like lantibiotics to promote colonization by more pathogenic skin bacteria such as Methicillin Resistant Staphylococcus Aureus (MRSA) and *Streptococcus pyogenes* (98–100). Furthermore, *S. epidermidis* strain-level diversity and their functional differences under distinct physiological conditions are increasingly being appreciated (79, 98, 101). For example, it has been suggested that disrupted barrier function (such as that seen in AD skin) can be exacerbated by *S. aureus* and promote the overgrowth of opportunistic *S. epidermidis* strains (98, 102). This hypothesis has been suggested in mouse studies where *S. epidermidis* is resistant to *S. aureus* challenge only in a healthy skin barrier state (103). In AD skin, *S. epidermidis* can injure the epidermis by producing the cysteine protease EcpA that can damage the skin when the organism achieves sufficient density to activate quorum-sensing mechanisms (104). Similarly, *S. epidermidis* cysteine protease EcpA is associated with disease in Netherton’s Syndrome and promotes skin damage through a disrupted barrier function (83). Though EcpA is part of the pan-genome of *S. epidermidis*, it is only expressed by some strains, further exemplifying the strain-level functional differences of *S. epidermidis* (104).

The characterization of flare *vs*. non-flare (AD-prone) lesions reveals the potential existence of an AD-susceptible microbiome profile marked by *Streptococcus* and *Gemella* enrichment and *Dermacoccus* depletion (105). Additionally, the function of fungi and viruses and the cross-kingdom interactions of AD microbiomes have yet to be completely investigated, as current studies have mostly concentrated on *S. aureus* and *S. epidermidis*. Differences in fungal and viral communities of the AD skin microbiome have also recently been observed and characterized by body site by Bjerre et al. (78). Increased *M. osloensis* and *M. luteu*s were observed in all AD skin sites except the neck, where it was absent. Perhaps unsurprisingly, AD lesions exhibit a higher abundance of *Staphylococcus* phages (78). According to these studies, dysbiosis in AD has global and body-site-specific differences involving bacteria, fungi, and viruses, and strain-level functional differences may contribute to disease complexities (**Table 1**).

### 5.2 Seborrheic dermatitis

Seborrheic dermatitis (SD) is another chronic skin disorder that causes a red, itchy rash with flaky skin (106). SD commonly occurs on the body’s oily areas like the scalp (where it is known as dandruff), face, and chest. Fungal and bacterial skin microbiome changes are currently implicated in SD, particularly in *Malassezia* and *Staphylococcus* species, with *Malassezia* associated with itchiness and disease severity (106–108). Topical antifungals (ketoconazole) are a common and sometimes effective therapy for SD. However, it is unclear if the mechanism of action occurs on microbes or other effects on the host from this class of drugs (109). To our knowledge, multiple studies have been conducted on the SD scalp microbiome using various 16S regions, and one study employs whole metagenomics. A recent systematic review by Tao et al. of all SD skin microbiome studies (largely conducted on the scalp) suggested an increased *M. restrica* to *M. globosa* and *Staphylococcus* to *Cutibacterium* ratios in SD skin (106). Only one study by Saxena et al. has performed a whole metagenome approach to study the functional pathways of SD and the skin microbiome. According to this study, bacterial alterations are characterized by increased *S. epidermidis*, decreased *P. nitroreducens*, and decreased *C. acnes* to *S. epidermidis* ratio (80). Fungal differences included increased *M. restricta* to *M. globosa* ratio and a significant portion of the mycobiome composed of uncharacterized *Malassezia* species and strains highly associated with dandruff presence (**Table 1**). Thus, SD represents an important example of how our understanding of the skin mycobiome is severely lacking (67) and how metagenomic assemblies of uncharacterized reads may resolve discoveries of new fungal species and strains.

### 5.3 Acne vulgaris

Acne is the most common inflammatory skin disease affecting approximately 9% of the population worldwide and 85% of teens and young adults (110). The visible nature of active acne and residual scarring in approximately 1 out of 5 disease cases creates psychosocial impacts through their negative effects on affected patients’ physical appearance, social interactions, and self-esteem. The increased incidence of anxiety, depression, and suicide arising from chronic acne, particularly for young adults, is well documented (111). Acne manifests in the pilosebaceous unit, commonly known as the hair follicle or “pore,” a lipid-rich niche on the skin with an acidic environment low in oxygen and nutrients that is hostile to most microorganisms (112). The skin microbiota is thought to play a causal role in acne development. In particular, there has been a focus on the role of the commensal skin bacterium *Cutibacterium acnes* (formerly named *Propionibacterium acnes*), an aerotolerant anaerobe that thrives in sebaceous conditions (64, 112). However, the link between *C. acnes* and acne disease has been challenging to pinpoint, even after decades of research and medical practice. The older model of acne implicated *C. acnes* skin “infection” as the disease’s cause due to its unique ability to thrive in the same environment where acne develops. This model proposed that increased sebum production in the pores promoted *C. acnes* overgrowth that physically “plugged” the hair follicle and sebaceous gland, thereby inducing inflammation in surrounding skin cells. Other work has suggested that disease in acne can come from cooperation between *C. acnes* and other inhabitants of the follicular microbiome, resulting in cell damage from a Christie, Atkins, Munch-Peterson (CAMP) reaction (113). Multiple studies have concluded that *C. acnes* is the predominant bacterium within most individuals’ skin microbial communities, both in healthy and diseased skin, and can account for nearly 90% of the skin pore microbiota, while its oxygen detoxification abilities allow it also to inhabit the skin surface (67, 79, 86). Nevertheless, topical and systemic antibiotics are a mainstay of acne therapy, and topical bacteriophage therapy has been suggested to have therapeutic potential (112, 114), suggesting that *C. acnes* has an important role in the pathophysiology of this disease.

As a commensal organism with a ubiquitous presence on the skin, the association of acne lesions with *C. acnes* is likely strain-dependent (115, 116). As researchers have attempted to understand how *C. acnes* subgroups are involved in acne disease, several different molecular typing methods have been developed to separate *C. acnes* into strains, making it difficult to cross-compare between studies (117). Genomic analyses concur specific strains of *C. acnes* are more commonly associated with acne (112, 116, 118, 119). However, the functional differences between *C. acnes* strains are generally unknown. It is even less clear if those strains that appear to be associated with acneic skin induce greater skin inflammation (and by what mechanisms). Furthermore, these current strain-typing methods are based on partial amplification and sequencing of conserved genes that likely fail to capture the full extent of *C. acnes* genetic diversity related to pathobiont behavior. For example, the first whole-genome sequencing analysis of *C. acnes* revealed the presence of a novel linear plasmid containing genes encoding potential virulence factors (120).

A greater understanding of the community compositional changes between acne and healthy skin is needed. In healthy skin, *C. acnes* and *S. epidermidis* are among the most prevalent commensals and have been shown to interact frequently with shifts in one species influencing the other. In acne-affected skin, the strain diversity and relative abundance of *S. epidermidis* are increased to inhibit the growth of *C. acnes via* fermentation and other defense mechanisms (112, 117, 121). A deep whole metagenome study by Barnard et al. of acne versus healthy individuals finds a balance of metagenomic elements shapes the skin microbiome in acne and health (81) (**Table 1**). Compared to healthy individuals, acne patients had a higher diversity of C. acnes populations, more strain types, more virulence-associated factors, and lower metabolic synthesis genes.

Additionally, their analyses suggest the *C. acnes* to *C. granulosum* ratio is important in acne disease, with acne patients having a lower relative *C. granulosum* abundance (81). *C. acnes* and *C. granulosum* have been reported to exist in a potentially competitive relationship, with *C. granulosum* secreting an endogenous extracellular nuclease capable of degrading *C. acnes* biofilm (122). Recently, a published study by Conwill et al. suggests *C. acnes* lineages can coexist across an individual’s skin; however, within individual pores, *C. acnes* is nearly clonal (< 1 mutation apart) (66). However, the *C. acnes* diversity at the single-pore level in individuals with acne has yet to be explored using per-pore sampling techniques.

### 5.4 Psoriasis

Psoriasis occurs in approximately 2% of the population worldwide and has characteristic plaques of red, itchy, and dry skin, most often on the elbows, knees, trunk, and scalp (123, 124). Many studies have shown dysbiotic differences in the psoriasis skin microbiome, especially in healthy and psoriatic individuals’ relative bacterial and fungal populations. While these associations are less clear than the previously mentioned diseases, many studies link *Streptococcus* and *Staphylococcus* bacterial species and *Malassezia* and *Candida albicans* fungi. Additionally, a subtype of psoriasis, known as guttate psoriasis, can be systemically triggered by bacterial infections such as “strep throat” from Group A *Streptococcus* (125). Psoriasis lesions have also been reported to have proportionately greater populations of *Staphylococcus. aureus* and *Streptococcus pyogenes* (126), and decreased incidence of *S. epidermidis* and *C. acnes* (82, 127). On psoriatic skin, *Malassezia* diversity decreases as disease severity increases. *M. restricta* and *M. globosa* are predominantly present, and *M. furfur* is reportedly only found on psoriatic skin. Another fungal microbe, *Candida albicans*, is also significantly more common in psoriatic patients and is linked to worsening psoriasis skin lesions (126, 127). In the first whole metagenome study of healthy versus psoriasis skin, Tett et al. suggest psoriatic lesions exhibit an increase in *Staphylococcus*; however, no significant differences were observed at the species level (82). However, their analyses suggest functional differences may lie at the strain level (**Table 1**). Additionally, similar to findings on the skin mycobiome in seborrheic dermatitis, the psoriasis mycobiome contained uncharacterized *Malassezia* reads, which the authors attributed to limitations in taxonomic databases.

### 5.5 Rosacea

Rosacea is a skin condition that causes facial flushing and sometimes small, pus-filled bumps on the face (71). The demographic of people most at risk for rosacea are individuals of Caucasian descent and who have fair skin, particularly women over 30 years of age. The skin microbiome is believed to be involved in rosacea; however, the results are inconsistent, likely due to mixed and limited methodologies (128). To the best of our knowledge, all current rosacea studies have used 16S amplicon sequencing and only used the V3 and V4 regions, which has been reported to be an issue in identifying *Staphylococcus* and *Cutibacterium* species (55). Whole metagenomics has not yet been employed to study the rosacea skin microbiome. Multiple studies have reported the skin microbiome of rosacea patients to have increased densities of *Demodex* mites, especially *D. folliculorum* and *D. brevis* (73, 129). (Characterization of *Demodex* mites is based on mitochondrial 16S rDNA partial sequences). While *Demodex* species are considered part of the healthy skin microbiome, in rosacea patients, an increased incidence of *Demodex* is associated with aberrant overactivation of the innate immune system (130). *Bacillus oleronius*, a bacterium carried by *Demodex*, can also independently trigger inflammatory pathways and may play a role in rosacea (73). Multiple studies also report a significant increase in the (normally) commensal bacterium *S. epidermidis* in rosacea patients, particularly within the pus-filled lesions (129, 131). At higher skin temperatures, *S. epidermidis* is known to produce additional proteins that may act as virulence factors (132). In rosacea, increased blood flow on the face leads to higher temperatures that may allow *S. epidermidis* to behave more opportunistically (129, 131). A decrease in *C. acnes* has also been noted in rosacea patients (133). Both *C. acnes* and *Demodex* mites live in the pilosebaceous unit and utilize sebum as a primary nutrient. Thus, the decrease in *C. acnes* may partly be due to the overgrowth of *Demodex*. However, many of these studies have not seen cross-validation. For a comprehensive review of the skin microbiome studies of rosacea, we direct the reader to the following (128).

### 5.6 Hidradenitis suppurativa

Also known as acne inversa, hidradenitis suppurativa (HS) is a chronic skin disease with painful nodule-like lesions that presents as abscesses and sinus tracts (tunnel-like wounds) on the skin (134). HS is considered an autoinflammatory disease caused by an aberrant and overactive innate immune system (134, 135). Flares from HS typically occur in body sites with more sweat glands and skin folding, such as the armpits, groin, buttocks, and breasts. Because of its appearance and location, HS can be misdiagnosed as acne, skin infection, herpes, or a sexually transmitted disease (STD). The psychosocial impacts of HS are significant due to its occurrence in private body sites, common misdiagnosis as a contagious STD, and permanent scarring, which is a common outcome. Current therapies are limited for HS, and this disorder is poorly responsive to antibiotics.

Several studies have recently characterized the skin microbiome of HS patients using 16S amplicon sequencing (136–142) (however, none with whole metagenome approaches). Across all studies, an increased abundance of anaerobic bacteria belonging to the *Porphyromonas* and *Prevotella* genera is significantly observed (138–142). Some studies also report an increase in *Corynebacterium* spp., *Moraxella* spp., *Actinomyces* spp., *Peptoniphilus* spp., *Mobiluncus* spp., and *Campylobacter ureolyticus* (138, 140, 142). In contrast, the skin of HS individuals has relatively lower populations of commensal bacteria, including *Cutibacterium* spp., *Staphylococcus epidermidis*, and *Staphylococcus hominis* (138, 142). Furthermore, some studies suggest an increased relative abundance of bacteria within lesional dermal tunnels versus non-lesional or non-dermal tunnel skin (142, 143). These findings demonstrate bacterial dysbiosis is associated with HS and varies with disease severity. Extending current findings with shotgun metagenomics will likely provide new insights into the skin microbiome’s role in this disease. Most bacteria currently associated with HS are resolved only at the genus level. An improved genomic analysis of the HS microbiome at the species or strain level will likely elucidate the association between this disease and the skin microbiome. There are currently no whole metagenome studies of the HS skin microbiome. For a comprehensive review of studies investigating the role of the skin microbiome in HS, we recommend the following (144).

### 5.7 Ichthyoses

Ichthyoses are a family of over 20 genetic skin diseases characterized by widespread, persistent dry, thick, and “fish-scale-like” skin (145). The various types of ichthyoses differ in their genetic inheritance pattern (which may be dominant, recessive, X-linked, or spontaneous), disease symptoms, and onset. Netherton Syndrome (NS) is an autosomal recessive-inherited type of ichthyosis where genetic mutations in the *SPINK5* gene results in the loss of function of lymphoepithelial Kazal-type-related serine protease inhibitor (LEKTI-1) (83). Although NS is a disease linked to germline mutation, its severity, and skin inflammation vary. One recent study investigated how the loss of LEKTI-1 influences resident skin bacteria and contributes to NS (83). Shotgun sequencing of the NS lesional skin microbiome demonstrated a dominance of *S. aureus* and *S. epidermidis*, both able to induce skin inflammation and barrier damage in mice (83). Due to increased proteolytic activity in the LEKTI-1 deficient state, these microbes promote skin inflammation *via S. aureus* phenol-soluble modulin α and increased bacterial proteases staphopain A and B from *S. aureus* or EcpA from *S. epidermidis*. Thus, NS presents an interesting case where a shotgun metagenomic approach plus experimental validation showed how a monogenic disease could influence microbial community shifts (**Table 1**).

## 6 Discussion

Just as culture-based studies helped us to discover microbial life on the skin, amplicon sequencing has greatly expanded our knowledge of previously unknown microorganisms. However, as newer technologies and analytical practices have become more refined, our previous goals of describing microbial skin communities at genus-level resolution are no longer a sufficient research aim, particularly when applied to understanding dermatological disorders. Amplicon-based approaches are still valuable tools, but studies employing them should utilize more resolved primer sets and current bioinformatics approaches to generate the most robust datasets possible. There is increasing awareness that sampling and analysis methods can significantly influence results as the skin exhibits multiple forms of variation by body-site-specific niches and between surface, follicular, and dermal layers. Additionally, improved and validated methods of reducing or depleting host DNA in skin samples will greatly improve skin microbial profiling. Thus, future studies should reassess some older conclusions and include further investigation of the subepidermal microbial distribution of specific skin diseases, which may provide new insights into how microbes may be modulating host physiology and immune responses. Additionally, we stress that the relationship between microorganisms and ourselves is often more nuanced than the commonly employed binary descriptions of commensal versus pathogenic. Many skin disorders are influenced by what we typically consider commensal microorganisms that appear to demonstrate pathological differences at the species or strain level. An improved understanding of higher-order taxa disease associations will likely clarify the involvement of microbes and support explaining the multifactorial nature and diverse manifestation of most skin diseases. Furthermore, an under-surveyed non-bacterial community consisting of fungi, viruses, skin mites, and their inter-species community relationships also appear to play an important role in skin diseases that we are only beginning to uncover.

With the rapid advancement of ‘omics’ technologies and improved accompanying bioinformatics methodologies, designing research studies to address these questions appropriately are now possible. Whole metagenomics profiling (even at shallow sequencing depths) yields richer datasets than amplicon-based sequencing; however, such studies are currently absent or limited in many skin diseases. Larger scale and systematic studies characterizing the healthy human skin microbiome, such as those aimed at understanding the human gut microbiome, will likely expand as the field progresses. Increasing metagenome-assembled genomes from skin-resident microorganisms (though still limited) are seeing greater development. Other methods, such as functional screening using transcriptomics, proteomics, and metabolomics, combined with profiling efforts, will offer a more comprehensive understanding of the functional potential of skin microbes toward disease and present gateways for clinical applications.

The causative relationship between changes in the skin microbiome and inflammatory skin diseases is a complex issue that remains a subject of debate in the field. While some skin diseases, such as atopic dermatitis, show clear links between microbiome dysregulation and disease onset, the degree of causation is less understood in other skin diseases. In most cases, however, it is likely that both dysregulated microbiome and alterations in the skin’s inflammatory environment play a dual role in disease pathogenesis. And whether which comes first may be different for different skin diseases. For example, *S. aureus* colonization has been shown to disrupt the skin barrier and overall skin health, contributing to enhanced colonization of opportunistic *S. epidermidis* strains and decreased overall commensal strains, which also negatively affect disease outcomes. Understanding the underlying mechanisms (as we have most clearly elucidated in atopic dermatitis) is critical to developing effective therapeutic interventions for skin diseases.

Future efforts to uncover the functional impact of microbial dysbiosis in the skin may also support an increased understanding of how the effects extend beyond the skin. We will likely continue to think less about microbiome ecosystems as being only confined to their anatomical location, as crosstalk mechanistic studies support such conclusions. There is increasing evidence for the systemic effects of skin microbiome dysbiosis and disease on distant microbial-related pathologies, termed the ‘skin-gut’ or ‘skin-brain’ axes. It is well documented that inflammatory skin disorders are frequently associated with inflammatory bowel disease and that many individuals with a skin disease also have comorbidity with a gut disease. Such relationships between a skin-gut axis have been reported for atopic dermatitis (146), acne (147), psoriasis (148), rosacea (71), and hidradenitis suppurativa (149). Additionally, in psoriasis, a skin-brain axis has been proposed, where skin cells that express neuropeptides and hormones may be directly promoting systemic inflammation and inducing psychological effects in the brain (150). To further explore the role of the skin microbiome in local and systemic health effects, experiments with new and improved clinically applicable models will be necessary to uncover the mechanisms. By continually evolving how we investigate microbes on the skin alongside rapidly advancing technological approaches, new findings push the frontier regarding our scientific knowledge of skin disease and whole-body health, providing opportunities to translate insights into clinical practice.

## Author contributions

YC, RK, and RG contributed to researching data and content. YC wrote the manuscript. YC, RK, and RG reviewed and edited the manuscript before submission. All authors contributed to the article and approved the submitted version.
